# Comparison effects of PEF and SC‐CO_2_ treatments on lycopene, β‐carotene, lutein, β‐cryptoxanthin, total polyphenols values, and antioxidant activity of tomato fruits

**DOI:** 10.1002/fsn3.4225

**Published:** 2024-08-29

**Authors:** Saba Belgheisi, Ali Motamedzadegan, Ladan Rashidi, Jafar M. Milani, Ali Rafe

**Affiliations:** ^1^ Department of Food, Halal and Agricultural Products Research Group Food Technology and Agricultural Products Research Center, Standard Research Institute (SRI) Karaj Iran; ^2^ Department of Food Science and Technology Sari Agricultural Sciences and Natural Resources University Sari Iran; ^3^ Department of Food Science and Technology Research Institute of Food Science and Technology Mashhad Iran

**Keywords:** bioactive compounds, extraction, pulsed electric field, supercritical fluid, tomato

## Abstract

Since the recycling of composites from plant tissues is difficult, extraction of bioactive compounds from plant sources requires pre‐treatment by new technology such as pulsed electric fields (PEF). Due to the reduced consumption of organic solvents, the extractive techniques such as using supercritical CO_2_ (SC‐CO_2_) are of interest to researchers. This work aimed to investigating the influences of different parameters of SC‐CO_2_ (pressure, modifier volume, temperature, and dynamic time) and PEF (frequency and field strength) treatments on the amount extraction of β‐carotene, lycopene, lutein, β‐cryptoxanthin, total phenol content (TPC), and also antioxidant activity percentage of tomato to obtain the optimum circumstances extraction via PEF and SC‐CO_2_ methods. PEF data showed that treatments with moderate intensity (1 Hz and 0. 25 kV/cm) enhanced the extractability of lycopene (88%), β‐carotene (69%), and β‐cryptoxanthin (24%). The maximum recovery in total polyphenols was achieved at a 1 Hz and 1.75 kV/cm, leading to a 41.68% growth. The SC‐CO_2_ results showed that extraction at 55°C and 35 MPa, and in a short time of 20 min (without any modifier: methanol) resulted in the highest levels of carotenoids (100% recovery), especially lycopene, and antioxidant activity. Largest value of total polyphenols was obtained at 35 MPa, 35°C, during 30 min, and 250 μL methanol as a modifier (58.79% recovery). Results showed that the extraction of polyphenols, unlike carotenoids, required a modifier. Organic solvents, often called modifiers, are sometimes added to the supercritical fluid to increase the polarity range of the extraction process and to help overcome analyte retention in the matrix. In this study, methanol was used as a modifier in different volumes. Therefore, the SC‐CO_2_ gentle processing conditions, compared with PEF, improved the recovery of tomato bioactive compounds and antioxidant activity. Nevertheless, further studies are needed to optimize such treatments.

## Graphical abstract

The SC‐CO2 moderate processing conditions improved extraction of tomato carotenoids. The PEF moderate processing conditions improved extraction of tomato carotenoids. The PEF at 1 Hz and 0.25 kV/cm increased carotenoids' extraction. The SC‐CO2 at 55°C, and 35 MPa, without modifier, achieved the highest carotenoids level.

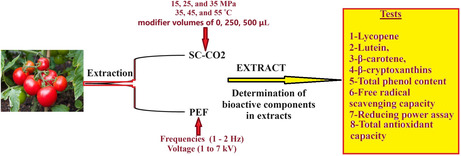

## 1 INTRODUCTION

The prominence of natural food additives has increased due to legal guidelines for using natural ingredients. For this reason, a great deal of articles has been published recently on extracting bioactive compound approaches especially carotenoids from natural resources (Keivanfar et al., 2023; Pataro et al., 2019; Strati & Oreopoulou, 2014). Tomato fruit is of interest to researchers due to the richness of carotenoid and polyphenolic compounds (Belgheisi et al., 2021; Motamedzadegan & Tabarestani, 2010). It is often difficult to recover such compounds from food because of physical and chemical barriers in the food structure. Therefore, a cell wall disruption technique like the usage of the pulsed electric field (PEF) method to extract beneficial compounds from food has been proposed (Pataro et al., 2019). This mechanism improves the permeability, termed “electroporation,” of the cytoplasmic membrane, which results in intracellular carotenoid composites being extracted (Kumar & Keum, 2018). So, the use of PEF at 0.5–80 kV/cm on plant samples induces electro‐permeabilization, leading to the higher extraction yield of metabolites of the plant (Buchmann & Mathys, 2019). PEF has been utilized to expand sucrose extraction from sugar beet, pigments like betaine from red beet, carotenoids from tomatoes, phenolic compounds from red wine, and oil from maize, as well as olive and rapeseed (Redondo et al., 2017). Different types of carotenoids with varied polarity levels make it difficult to extract them simultaneously. Moreover, the exposure to excess light, heat, acids, and long extraction times is limited by the carotenoids’ oxidative features. Carotenoids are usually extracted using organic solvents which are generally expensive, toxic, and dangerous to control and may persevere in the product (Belgheisi et al., 2021; Kumar & Keum, 2018). Supercritical fluid extraction (SFE) is a suitable technique for extracting thermolabile compounds like carotenoids, using supercritical CO_2_ as the extracting solvent. Because of the non‐polar nature, carbon dioxide has been applied successfully to non‐polar compounds extraction from plants (Madia et al., 2021). It is possible to adjust a supercritical fluid's solvent strength, which is directly associated with its density, by varying the temperature and pressure during extraction (Topal et al., 2006). The use of supercritical carbon dioxide has been studied by several authors for extracting carotenoids from some matrices, such as tomatoes (Cadoni et al., 2000; Rozzi et al., 2002; Sabio et al., 2003; Shi et al., 2009; Vagi et al., 2007; Vasapollo et al., 2004). The extraction from tomato and its product via SFE was limited to lycopene and β‐carotene, and more studies have not been reported. Also, there is a little investigation designed to assess the impact of PEF treatments under different conditions (including frequency and electric field strength) on the tomato bioactive compound extraction. The aim of this study was to determine the optimum values of the different factors like frequency and electric field strength in the PEF process and also temperature, pressure, dynamic extraction time, and modifier volume involved in the SFE process using chemometric tools (Taguchi), which were designed to obtain the higher recovery of β‐carotene, lycopene, β‐cryptoxanthin, lutein, and polyphenols from tomato fruit (determined by the HPLC‐UV). In addition, the antioxidant activity of extracts under different conditions was determined using DPPH, RPA, and TAC.

## 2 MATERIALS AND METHODS

### 2.1 Chemicals and reagents

Acetonitrile, acetone, methanol, and hexane with HPLC grade have been purchased from Fisher Scientific (Fair Lawn). DPPH (2,2‐diphenyl‐1picrylhydrazyl), Folin–Ciocalteu thiosulfate reagents, and the standard reference materials of lycopene, β‐carotene, lutein, and β–cryptoxanthine were obtained from the Sigma‐Aldrich company. Moreover, gallic and ascorbic acids were obtained from Merck.

### 2.2 Preparation of sample

The tomato fruits (*Solanum Lycopersicon L*.) were obtained from the Rojin Crop Industry in Kermanshah, western Iran, in March–July 2018. The cultivars H1015 (Heinz) were studied in this research. Tomato fruits were harvested by hand at the red‐ripe (over 90% red surface) maturity phase (an average weight of 210–250 g). A minimum of 2 kg of the damage‐free tomato fruit was transported to the laboratory. The tomato fruits have been sliced into four parts and chopped with a homemade blender (DP705, Moulinex).

For SC‐CO_2_ treatment, the tomato puree is dried in a vacuum drier (5831, Napco) at 40°C for 24 h until the moisture reaches 10% (w/w), then ground with a household grinder (DP705, Moulinex) to obtain the fine particle size of 0. 5–1 mm, and then the powder stored at −20°C.

### 2.3 PEF treatment

Through a batch mode PEF mechanism (PEF, RIFST), the treatments were conducted at the Research Institute of Food Science and Technology, Mashhad, Iran. A sample size of 250 g of tomato puree was used for every PEF run. The sample was put and handled in a parallel electrode batch treatment chamber with a breach of 4 cm in the surface electrode zone of 16 cm^2^. The device produced square waveform pulses by frequencies of 1 and 2 Hz in bipolar. The peak pulse voltage varied between 1 and 7 kV, which creates electric field strengths ranging from 0.25 to 1.75 kV/cm. At ambient temperature, 30 pulses of a pulse width 20 μs were used. With increasing the pulse width (τ) through the number of used pulses consistent with values 0.6 ms, the treatment time was estimated. Table 1 illustrates the PEF treatment conditions. The PEF treatment conditions were designed by Taguchi. The sample primary temperature in all PEF experiments was 20 ± 1°C, and due to the low‐energy input transported within the treatment, there was no considerable temperature rise. Before the analysis, PEF‐treated and untreated tomatoes were kept at −20°C for 24 h, instantly.

**TABLE 1:** PEF treatment conditions.

Run	Frequency (Hz)	Voltage (kV)	Electric field strength(kV/cm)	Time (ms)
1	1	1	0.25	0.6
2	1	4	1	0.6
3	1	7	1.75	0.6
4	2	1	0.25	0.6
5	2	4	1	0.6
6	2	7	1.75	0.6

### 2.4 SC‐CO_2_ treatment

A Suprex MPS/225 system (Pittsburgh, PA) in the SFE mode was applied for all the extractions. A stainless‐steel vessel (10 mL) was used as an extraction cell. To gather the extracted analytes, a Dura flow manual variable restrictor (Suprex), maintaining the supercritical pressure conditions in the system and adjusting the CO_2_ flow rate, was utilized in the SFE system. In the dynamic extraction phase, a supercritical fluid flow rate of 0.30 ± 0.05 mL/min was applied, and the restrictor point was heated electrically to avoid plugging the sample. The extraction vessel was packed with 2.5 g of tomato powder. Nine extractions were performed at three temperatures of 35, 45, and 55°C, three pressures of 15, 25, and 35 MPa, and three modifier volumes of 0, 250, and 500 μL within 15 min of static extraction after 10, 20, and 30 min of dynamic extraction. The SFE experimental conditions for bioactive compound extraction of tomato (according to the Taguchi) are presented in Table 2. Then, extracted analytes were gathered in a 5.0 mL volumetric flask containing hexane solvent. The ultimate extract volume was set to 5.0 mL with the hexane at the final extraction. Then, modifier was added by pipetting methanol and dropping the modifier to the specimen in an extraction cell before the cell was connected to the SFE system. After each run, 5 mL of the gathered solution was purred into a specimen vial equipped with a septum for analysis.

**TABLE 2:** SC‐CO_2_ treatment conditions.

Run	Pressure (MPa)	Temperature (°C)	Dynamic time (min)	Modifier volume (μl)
1	15	35	10	0
2	15	35	20	250
3	15	35	30	500
4	25	45	20	500
5	25	45	30	0
6	25	45	10	250
7	35	55	30	250
8	35	55	10	500
9	35	55	20	0

### 2.5 Lycopene, lutein, β‐carotene, and β‐cryptoxanthin contents determination

The HPLC (Young Lin 9100) equipped with a UV–Vis detector, the LiChrospher® RP‐18 HPLC Column (25 cm × 4 mm, packed with 4 μm particle size) and also the mobile phase (methanol: THF: water (67:27:6)) with flowrate 2 mL/min were used for determination of lycopene, lutein, β‐carotene, and β‐cryptoxanthin, based on the described method by Sadler et al. (1990) as adapted by Perkins‐Veazie et al. (2001). Outcomes were obtained based on mg/100 g fresh fruit weight (fw).

### 2.6 Total polyphenols content (TPC) determination

TPC was measured according to the method described by Kazempour‐Samak et al. (2021); Yousefi et al., 2023. 0.5 mL of Folin–Ciocalteu reagent (10%w/v) was added to 0.1 mL of diluted extract (0.01 g in 10 mL of ethanol), and after 5 minutes, sodium carbonate (0.4 mL, 7.5%) was added. The absorbance of the sample against the distilled water control at 765 nm was read using a spectrophotometer (PerkinElmer Lambda 25 UV/Vis Spectrometer) after 30 min of incubation at room temperature. The concentration of TPC was determined based on the formula (y = 0. 396 x − 0. 364, *R* ^2^ = . 999) obtained from the standard curve of gallic acid (50–500 μg/mL).

### 2.7 Free radical scavenging capacity (DPPH assay)

First, 100 μL of methanolic DPPH solution (0.5 mM) was added to 100 μL of extract. Then, 1 mL of methanol was added to it and mixed well. The solution was stored in darkness for 30 min at room temperature. Then, the absorbance of the sample was measured at 517 nm. Ascorbic acid was applied as a positive control (Kazempour‐Samak et al., 2021; Yousefi et al., 2023). The free radical scavenging capacity was calculated as follows:

$$\mathrm{AA}\;\left(\%\right)=\left(\mathrm{Ac}-\mathrm{As}\right)/\mathrm{Ac}\times 100$$

Ac: absorbance of the control sample.

As: absorbance of the sample

### 2.8 Reducing power assay (RP)

Based on a method described by El Jemli et al. (2016), 0.2 mL of the extract at various concentrations with 2.5 mL of phosphate buffer (0.2 M) at pH 6.5 and 2.5 mL of potassium ferricyanide K3Fe(CN)6 (1%) were mixed and then incubated at 50°C for 20 min. Then, 2.5 mL of 10% (w/v) trichloroacetic acid was added and centrifuged at 1000 rpm for 10 min. In the end, the upper layer (2.5 mL) was mixed with distilled water (2.5 mL) and 0.5 mL of FeCl3 (0.1%). The absorbance was determined at 700 nm. A standard curve was obtained using the ascorbic acid at different 50–300 μg/mL concentrations. The calibration curve equation was obtained y = 0. 011x–0. 285, *R* ^2^ = . 975.

### 2.9 Total antioxidant capacity (TAC)

Measuring the total antioxidant capacity was based on the technique explained by Prieto et al. (1999). 0.5 mL of the extract was mixed with 5 mL of phosphomolybdate reagent (a mixture of 0.6 M sulfuric acid, 28 mM sodium phosphate, and 4 mM ammonium molybdate), and then it was kept in a 95°C water bath for 90 min. After the resulting mixture was cooled to room temperature, the absorbance at 695 nm wavelength was read against a control sample. The antioxidant capacity of all extracts is reported in mg of ascorbic acid per 100 g of tomato sample. Different concentrations of ascorbic acid were used to draw a calibration curve to measure total antioxidant activity.

(50–300 μg/mL, y = 0.011x–0. 325, *R* ^2^ = . 981)

### 2.10 Experimental design and statistical analysis

Experimental design was done by Taguchi method, with Minitab software version 16.

All experiments were performed in triplicate. Statistical treatment analysis was done using the variance analysis (ANOVA) SPSS software version 22. The Duncan test determined a significant difference between means at 0.05.

## 3 RESULTS AND DISCUSSION

### 3.1 The influence of PEF and SC‐CO_2_ methods on carotenoids and total polyphenols content extraction

The use of PEF and SC‐CO_2_ treatments increased considerably (*p* < .05) the amounts of lycopene, β‐carotene, lutein, and β‐cryptoxanthin taken out from tomato fruit (Table 3). The data of PEF treatment showed that at 1 Hz and 0.25 kV/cm (0.6 ms − 30 pulses) (run1), resulting in an 88%, 69%, and 24% increase in lycopene, β‐carotene, and β‐cryptoxanthin, respectively, in comparison to the untreated fruit content. Lutein concentration increased by 37% once tomatoes were treated at 1 Hz and 1.75 kV/cm (0.6 ms–30 pulses) (run3).

**TABLE 3:** The means of carotenoids and total polyphenols content extracted from tomatoes under different PEF conditions.

Treatments	Lycopene (mg/100 g)	b‐carotene (mg/100 g)	Lutein (mg/100 g)	b‐cryptoxanthin (mg/100 g)	TPC (mg/100 g as gallic acid)
Control	12.018 ± 0. 05^e^	3.456 ± 0.05^e^	0.244 ± 0.006^e^	0.365 ± 0.05^e^	7.33 ± 0.006^g^
Run1	22.579 ± 0.052^b^	5.826 ± 0.05^a^	0.295 ± 0.005^c^	0.453 ± 0.005^a^	11.53 ± 0.006^d^
Run 2	15.270 ± 0.05^c^	5.237 ± 0.05^b^	0.307 ± 0.025^b^	0.430 ± 0.05^b^	12.28 ± 0.01^b^
Run 3	12.948 ± 0.01^d^	4.019 ± 0.01^d^	0.334 ± 0.005^d^	0.240 ± 0.015^g^	12.57 ± 0.023^a^
Run 4	10.618 ± 0.05^f^	4.438 ± 0.05^c^	0.249 ± 0.001^d^	0.385 ± 0.005^c^	8.91 ± 0.01^f^
Run 5	10.508 ± 0.05^g^	2.498 ± 0.02^f^	0.201 ± 0.005^g^	0.372 ± 0.005^d^	10.19 ± 0.01^e^
Run 6	4.497 ± 0.042^a^	1.728 ± 0.005^g^	0.222 ± 0.005^f^	0.328 ± 0.005^f^	11.95 ± 0.006^c^

*Note*: Values with different letters in each column are significantly different (*p* < .05).

Values are means ± S.D. of three independent experiments.

The codes of each treatment are according to Table 1 (PEF treatment conditions).

The results revealed that PEF had the most affected on lycopene and β‐carotene extraction. Increasing the frequency and electric field strength reduced the amounts of extractive pigments except for lutein, which was raised by an increase in the power of the electric field from 0.25 kV/cm to 1.75 kV/cm at 1 Hz. Such information reveals that the PEF may function unpredictably. The same phenomenon was observed in the studies of Vallverdu‐Queralt et al. (2015); they established that by rising electric field strength, the amount of lycopene as well as total carotenoids reduced, which is possibly due to the irretrievable electroporation of cell membranes. Pataro et al. (2019) applied PEF treatment on tomato peel before extraction by acetone at various temperatures up to 50°C compared to untreated samples; the optimal energy input was 5 KJ/Kg at the field strength 5 kV/cm increased the amount of the total carotenoids. By HPLC analysis, no isomerization or decomposition of carotenoid content occurred.

Also, this study was in line with the results of Wiktor et al. (2015), which assessed the PEF treatment impact on some bioactive compounds and determined that by increasing the strength of the electric field from 1.85 kV/cm to 3 kV/cm, the amounts of carotenoid compounds extracted from fruit tissues were reduced. Rising the carotenoid concentration in low‐frequency (1 Hz) and low‐voltage (1 kV) was associated with activating the metabolic pathway of carotenoids and increasing the extraction ability from food due to the cell membrane penetrability. Reducing the value of carotenoids extracted by increasing the frequency and voltage can be attributed to the cell membranes' irreversible electroporation (Gonzalez_Cusado et al., 2018), their reaction with reactive oxygen species, which is formed due to the cell response to stress, induced by electric current (Botero‐Uribe et al., 2017). The amounts of carotenoids extracted by SC‐CO_2_ treatments are shown in Table 4.

**TABLE 4:** The means of carotenoids and total polyphenols content extracted from tomatoes under different SC‐CO_2_ conditions.

Treatments	Lycopene (mg/100 g)	b‐carotene (mg/100 g)	Lutein (mg/100 g)	b‐cryptoxanthin (mg/100 g)	TPC (mg/100 g as gallic acid)
Control	12.018 ± 0.05^b^	3.456 ± 0.05^a^	0.244 ± 0.006^c^	0.365 ± 0.05^a^	7.33 ± 0.006^c^
Run 1	N.D	0.1054 ± 0.006^j^	0.059 ± 0.05^j^	0.0701 ± 0.005^j^	6.09 ± 0.01^f^
Run 2	2.199 ± 0.05^e^	0.115 ± 0.05^i^	0.135 ± 0.001^g^	0.222 ± 0.001^g^	7.36 ± 0.01^b^
Run 3	12.559 ± 0.05^a^	0.456 ± 0.05^e^	0.255 ± 0.05^b^	0.292 ± 0.05^b^	5.78 ± 0.01^i^
Run 4	2.838 ± 0.004^d^	0.198 ± 0.006^f^	0.194 ± 0.05^e^	0.091 ± 0.03^i^	5.84 ± 0.02^h^
Run 5	4.415 ± 0.05^c^	2.962 ± 0.05^b^	0.285 ± 0.002^a^	0.268 ± 0.05^d^	7.22 ± 0.01^d^
Run 6	1.522 ± 0.01^f^	0.124 ± 0.05^h^	0.115 ± 0.05^i^	0.197 ± 0.05^h^	5.88 ± 0.006^g^
Run 7	2.739 ± 0.05^d^	0.515 ± 0.001^d^	0.135 ± 0.05^h^	0.241 ± 0.001^e^	7.39 ± 0.01^a^
Run 8	2.881 ± 0.05^d^	0.186 ± 0.005^g^	0.162 ± 0.001^f^	0.228 ± 0.05^f^	6.92 ± 0.01^e^
Run 9	12.737 ± 0.05^a^	1.68 ± 0.01^c^	0.229 ± 0.006^d^	0.285 ± 0.05^c^	5.66 ± 0.01^j^

*Note*: Values with different letters are significantly different (*p* < .05).

Values are means ± S.D. of three independent experiments.

The codes of each treatment are according to Table 2 (SC‐CO_2_ treatment conditions).

Lycopene was not observed in chromatographic analysis after treatment at low pressure and temperature (run 1). Cadoni et al. (2000) stated that lycopene extraction at 40°C and at pressures less than 25 MPa was truly insignificant. The most significant amounts of lycopene and β‐cryptoxanthin were detected at 35 MPa and a temperature of 550°C for 20 min, using any modifier (100% recovery).

The highest recovery of β‐carotene (85.7%) and lutein (100%) was obtained at 25 MPa and 45°C for 30 min, without any modifier. Oppositely, increasing the temperature from 35°C increased the solute partial pressure, as a result of the volatility influence, as well as enhancing mass transfer. Increasing the extraction temperature to 55°C resulted in an easy partitioning procedure by increasing the main components' vapor pressure. More significant pressures are needed to complete the plant's components recovery. So, increasing the pressure from 15 MPa to 35 MPa improved the extraction recovery due to greater fluid density caused by increasing the extraction pressure at a constant temperature, increasing the solubility of analytes. Mihalcea et al. (2021) extracted lycopene‐rich oleoresin from tomato peels using SC‐CO_2_ treatment which resulted in high amounts of lycopene‐enriched oleoresin at 74°C, and 40 MPa during 155 min. The same results were reported by Kehili et al. (2017). Topal et al. (2006) concluded that increasing temperature and pressure improved the lycopene extracted from tomato skin with SC‐CO_2_. Increased extraction pressure increases the yield in lycopene recovery (Lenucci et al., 2010; Rozzi et al., 2002); however, applications using the pressure values within the range of 20 MPa–45 MPa create optimal results relying on other procedure parameters, particularly extraction temperature and product's features and type (Konar et al., 2012). The use of PEF and SC‐CO_2_ treatments touched meaningfully (*p* < . 05) the amount of TPC in tomato fruit (Table 4). The PEF treatment showed that increasing the electric field strength from 0.25 kV/cm to 1.75 kV/cm enhanced the amount of total polyphenols; however, increasing the frequency from 1 Hz to 2 Hz reduced the amount of total polyphenol. The maximum enrichment in total polyphenols was achieved in tomatoes exposed to treatments at a frequency of 1 Hz and electric field strength of 1.75 kV/cm (0. 6 ms–30 pulses), leading to a 41.68% growth compared with the untreated fruit content. According to the studies, it has been demonstrated that the use of PEF with high electric field strength may motivate a considerable decrease in the enzyme's activity, like polyphenol oxidase, lipoxygenase, and pectin methyl esterase. Structural changes due to PEF may help the extraction of bioactive composites like polyphenols, as Puertolas et al. (2010) and Luengo et al. (2013) showed the same results. Our studies contradicted the results of Wiktor et al. (2015), which concluded that as electric field strength improved, the quantity of polyphenols extracted from apple peel decreased. Table 3 shows the quantity of total polyphenols extracted by SC‐CO_2_ treatments. The most significant quantity of total polyphenols was detected at 35 MPa, 35°C, during 30 min, and 250 μL modifier (58. 79% recovery). Extraction of polyphenols, unlike carotenoids, required a modifier. Without the co‐solvent, the amounts of recycled polyphenols were from 45% to 48%. Polyphenols are fairly polar compounds, and when extracted by SC‐CO_2_ a modifier normally is inserted to make a further polar environment and increment the target compounds' solubility in the supercritical solvent. Adding methanol raises the SC‐CO_2_ critical temperature. Introducing methanol improves the SC‐CO_2_ solvent power, leading to enlargement in the matrix, increasing the internal volume and the surface area, as well as decomposing the cellular wall, thus probably enhancing the bioavailability of polyphenols for extraction. Consequently, very excellent yields have been reported for the modifier‐supported SC‐CO_2_ extraction of polyphenols from by‐products. Casas et al. (2010), for instance, reported that resveratrol, in grape by‐products, was extracted at higher pressure (40 MPa) and lower temperature (35°C) by 5% v/v ethanol as a co‐solvent. The highest quantity of polyphenolic compounds was recovered at 35°C; this is lower compared to the optimal conditions to some extent for carotenoid extraction and would seem to imply that compared to carotenoids, polyphenols are less heat‐stable. Like carotenoids’ extraction, the pressure of 35 MPa was optimal for polyphenols’ extraction. However, SC‐CO_2_ produced lower phenolic concentrations than the traditional extraction technique (Herrero et al., 2010).

### 3.2 The impact of PEF and SC‐CO_2_ treatments on antioxidant activity

**FIGURE 1:**
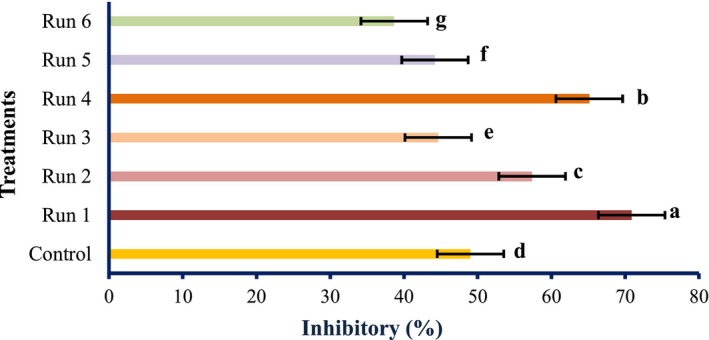
DPPH radical scavenging activity of tomatoes treated with PEF (values are means ± S.D. of three independent experiments, values with different letters are significantly different (*p* < .05)). The codes of each treatment are according to Table 1 (PEF treatment conditions).

**FIGURE 2:**
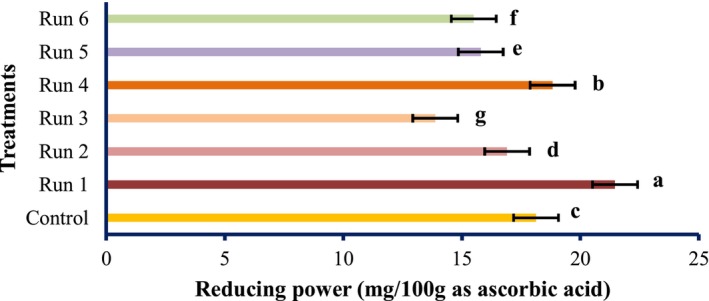
Reducing power of tomatoes treated with PEF (values are means ± S.D. of three independent experiments, values with different letters are significantly different (*p* < .05)). The codes of each treatment are according to Table 1 (PEF treatment conditions).

**FIGURE 3:**
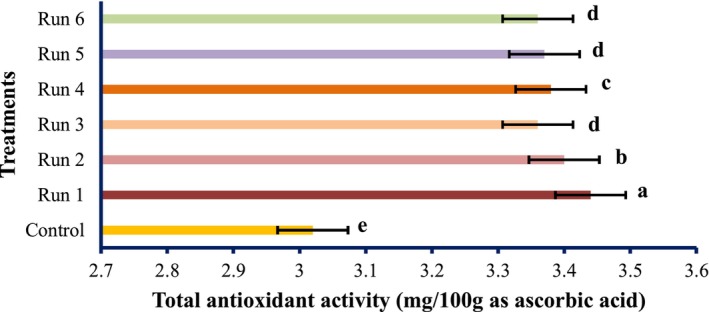
Total antioxidant activity of tomatoes treated with PEF (values are means ± S.D. of three independent experiments, values with different letters are significantly different (*p* < .05)). The codes of each treatment are according to Table 1 (PEF treatment conditions).

The antioxidant activities of PEF‐treated tomato samples are shown in Figures 1, 2, 3.

**FIGURE 4:**
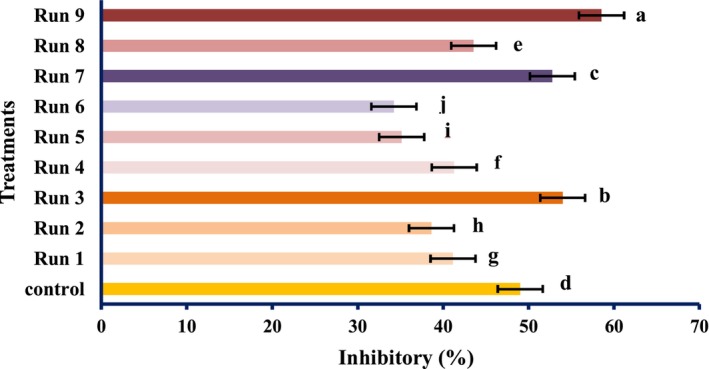
DPPH radical scavenging activity of tomatoes treated with SC‐CO_2_ (values are means ± S.D. of three independent experiments, values with different letters are significantly different (*p* < .05)). The codes of each treatment are according to Table 2 (SC‐CO_2_ treatment conditions).

**FIGURE 5:**
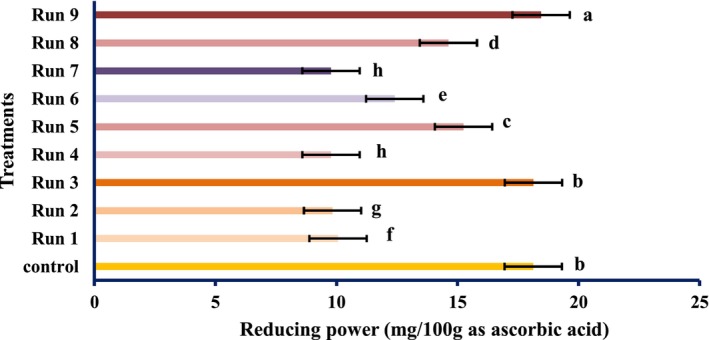
Reducing power of tomatoes treated with SC‐CO_2_ (values are means ± S.D. of three independent experiments, values with different letters are significantly different (*p* < .05)). The codes of each treatment are according to Table 2 (SC‐CO_2_ treatment conditions).

**FIGURE 6:**
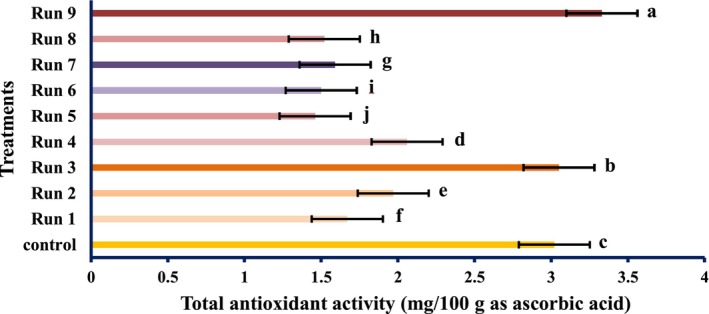
Total antioxidant activity of tomatoes treated with SC‐CO_2_ (values are means ± S.D. of three independent experiments, values with different letters are significantly different (*p* < .05)). The codes of each treatment are according to Table 2 (SC‐CO_2_ treatment conditions).

The radical scavenging activity of the PEF‐treated samples varied remarkably (*p* < .05) and increased in the untreated samples. Although the total antioxidant activity and reducing power of PEF‐treated samples differed meaningfully (*p* < .05), however, it decreased by PEF treatments compared with the untreated specimen. However, the highest antioxidant activity was obtained at 1 Hz and 0.25 kV/cm electric field strength. At constant frequencies, the strength of the electric field of 0.25 kV/cm showed the highest antioxidant activity, which was reduced by increasing the electric field strength. Therefore, it can be concluded that PEF can boost the radical scavenging activity of natural compounds. These findings were in line with the results of Wiktor et al. (2015), which showed that antioxidant activity increased by 90% in grape peel treated with PEF. Corrales et al. (2008) showed that the electric field strength use of 3 kV/cm raises the compounds and the antioxidant activities as per the DPPH assay of red grapes. However, our data contradicted the study of Pataro et al. (2019); they found that the extracts obtained from tomato peels treated with PEF had higher antioxidant activity than the control, and with increasing field strength, the antioxidant activity was higher. It can be decided; thus, PEF treatment has an unexpected effect on the antioxidant action of tomato fruit, which may be accredited to the different treatment conditions, analytical methods, fruit type, and variety. The impact of SC‐CO_2_ conditions on the antioxidant activity of tomato extract is shown in Figures 4, 5, 6.

The extract with the highest antioxidant activity (DPPH, decreasing power, and total antioxidant activity assays) was the one obtained at 55°C, 35 MPa, 20 min, with no modifier. According to Kehili et al. (2017), the greatest DPPH quenching activity of 84.51 ± 5.25% was found at low temperature of 50°C and 30 MPa pressure. As anticipated, mild extraction circumstances may result in better maintenance of antiradical activity. Egydio et al. (2010) reported that at the low temperature of 40°C, the most significant DPPH antiradical activity value was obtained, while keeping the supercritical CO_2_ pressure at 35 MPa. Similarly, Liu et al. (2011) stated that at low extraction pressure, the pomegranate seed oil extraction (15 MPa) proved greater antioxidant capability in contradiction of DPPH radical compared to higher pressure (30 MPa), while the higher pressure encouraged a pomegranate seed oils higher extraction yield. According to Martinez‐ValverdeI et al. (2002), and Takeoka et al. (2001) studies, the main compound in charge of repossessing free radicals was lycopene, and the extract's greater antioxidant capacity found at 55°C and 35 MPa could be ascribed to the existence of higher quantities of this compound in the extract found in this particular circumstance. In general, the change in antioxidant activity is mostly affected by their lycopene content. Mihalcea et al. (2021) reported that at a pressure of 40 MPa and the CO_2_ flow of 0.32 kg/min for temperatures of 70°C, 74°C, and 80°C, the maximum antioxidant activity was obtained in 74°C lycopene extraction from tomato peels using SC‐CO_2_ treatment.

## 4 CONCLUSION

The correct selection of PEF and SC‐CO_2_ treatment parameters can improve the ability to extract bioactive compounds in tomato fruit. The use of moderate‐intensity PEF (0.25 kv/cm) has beneficial effects because it increases the amount of some bioactive compounds like carotenoids with high antioxidant activity (radical scavenging activity) in the array of field strength investigated. To find the best extraction conditions, the results revealed that the SC‐CO_2_ extraction treatments of carotenoids and polyphenols require quite different factors in temperature, pressure, the volume of the modifier, and dynamic time. Under moderate processing conditions, we were able to achieve the highest recovery of bioactive compounds and antioxidant activity. Also, optimizing extraction conditions toward the loss consumption of organic solvent with the maximum extraction recovery is supreme from the extract quality and processing cost. The present research showed that the PEF and SC‐CO_2_ treatments have the potential to enhance tomato pigments extraction, but PEF is more cost‐effective due to less consumption of energy and organic solvents. However, more research is needed to endorse the use of this equipment on an industrial scale by examining the different process conditions and the use of other tomato cultivars.

## AUTHOR CONTRIBUTIONS

**Saba Belgheisi:** Formal analysis (equal); writing – original draft (equal). **Ali Motamedzadegan:** Supervision (equal); writing – review and editing (equal). **Ladan Rashidi:** Conceptualization (equal); writing – review and editing (equal). **Jafar M. Milani:** Software (equal); visualization (equal). **Ali Rafe:** Methodology (equal); writing – review and editing (equal).

## FUNDING INFORMATION

All authors declare that no funds and grants were received during this research.

## CONFLICT OF INTEREST STATEMENT

There are no conflicts of interest with respect to the research described in this manuscript.

## Data Availability

The current study is available from the corresponding author upon reasonable request.
